# Infectious seeds of valve calcification: Exploring the bacterial hypothesis in the pathogenesis of calcific aortic valve disease

**DOI:** 10.1111/eci.70188

**Published:** 2026-03-08

**Authors:** Antonella Forlino, Paola Giordani, Cristina Merla, Silvia Roda, Roberta Besio, Abeer Ahmed Qaed Ahmed, Angela Kuka, Irene Mileto, Greta Petazzoni, Marta Corbella, Patrizia Cambieri, Baldanti Fausto, Annalisa De Silvestri, Totaro Pasquale, Filippo Amoroso, Pelenghi Stefano, Maraschi Federica, Eloisa Arbustini, Viviana Vilardo, Alexandra Smirnova, Raffaele Bruno, Seminari Elena

**Affiliations:** ^1^ Department of Molecular Medicine, Biochemistry Unit University of Pavia Pavia Italy; ^2^ Biostatistics and Clinical Epidemiology, Fondazione IRCCS Policlinico san Matteo Pavia Italy; ^3^ Malattie Infettive, Fondazione IRCCS Policlinico San Matteo Pavia Italy; ^4^ Microbiologia e Virologia, Fondazione IRCCS Policlinico San Matteo Pavia Italy; ^5^ Infectious Diseases Unit Fondazione IRCCS Ca’ Granda Ospedale Maggiore Policlinico Milan Italy; ^6^ Cardiac Surgery Unit Fondazione IRCCS Policlinico san Matteo Pavia Italy; ^7^ Department of Chemistry University of Pavia Pavia Italy; ^8^ Department of Research, Centre for Inherited Cardiovascular Diseases Fondazione IRCCS Policlinico San Matteo Pavia Italy; ^9^ Department of Medicine, Surgical, Diagnostic and Pediatric Science University of Pavia Pavia Italy

**Keywords:** calcific aortic valve disease, osteoblast, osterix, staphylococcus spp., streptococcus spp., VIC

## Abstract

**Background:**

Calcific aortic valve disease (CAVD) is a multifactorial condition characterized by progressive leaflet calcification with a potential role for bacterial colonization in its pathogenesis. This study investigates clinical, microbiological and molecular features of calcified versus non‐calcified aortic regurgitation (AR) valves.

**Methods:**

This is a prospective, observational study, whose primary objective was to compare the occurrence of bacterial detection between CAVD and AR. The secondary objectives included the evaluation of bone‐related calcification markers in valves from CAVD and AR patients.

**Results:**

We analysed 31 CAVD and 8 AR valves, yielding 111 leaflets (84 calcified, 27 non‐calcified). Light microscopy of CAVD leaflets revealed near‐complete disruption of the three‐layered valve architecture, with calcified masses extending through the leaflets, sparse cellularity and focal micro‐angiogenesis; no bacteria were detected by GRAM, PAS or TEM. Enrichment culture detected low‐virulence bacteria in 5.95% of CAVD and 4.16% of AR leaflets; 16S rRNA PCR was positive in 22.5% of CAVD and 12.5% of AR cases, with *Staphylococcus* and *Streptococcus* spp. predominating. Calcium content was significantly higher in CAVD leaflets (*p* = .001) and correlated with dyslipidemia (*p* = .02). Osterix expression was higher in valves with positive microbiological findings (*p* < .0001), while ALP was increased in CAVD and bicuspid valves regardless of microbial status. Valve interstitial cells from CAVD exhibited spontaneous in vitro calcification, unlike controls.

**Conclusion:**

The early osteogenic marker osterix was found to be upregulated in patients whose valves tested positive for microbial DNA, suggesting a potential role for bacteria in driving cellular differentiation towards an osteoblastic phenotype in CAVD.

## Graphical abstract

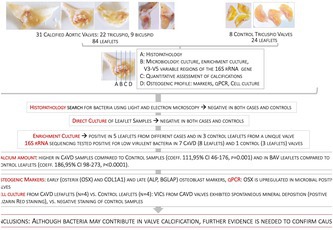

## 1 INTRODUCTION

Calcific aortic valve disease (CAVD) is highly prevalent worldwide, affecting nearly 50% of individuals over the age of 85. CAVD is characterized by slowly progressive fibro‐calcific remodelling of the valve leaflets causing aortic stenosis.^1^ Risk factors for CAVD partly overlap those for atherosclerosis, but also include age‐related tissue changes and the effects of comorbidities (i.e. renal failure).^2^, ^3^, ^4^ The spectrum of the disease progression starts with leaflet degeneration and progresses from early lesions to valve stenosis/obstruction.^1^, ^5^ Calcific aortic stenosis is characterized by osteogenic metaplasia with osteoblast‐like cells and chondrocytes associated with dense inflammatory infiltrates.^6^ Bacteria have been detected in calcific aortic valves in the absence of diagnosis of acute infective endocarditis, but their role in the pathogenesis remains unclear.^7^, ^8^ Indeed, it has been recently demonstrated that bacterial infections can directly affect osteoblast differentiation/activation.^9^

This study aimed to assess whether calcified aortic valves harbour bacterial infiltration and to explore its potential correlation with ectopic calcification and the upregulation of osteogenic markers.

## 2 METHODS

### 2.1 Design of the study

This is a prospective, observational study conducted at Fondazione IRCCS Policlinico San Matteo between 15 May 2023 and 31 July 2024.

To investigate the infective, biochemical and structural features of CAVD, we established a multidisciplinary team comprehensively addressing the clinical, histological, cellular, genetic and molecular features.

The case series was constituted of native aortic valves removed from patients with CAVD (stage III and IV) who underwent aortic valve replacement in accordance with International Guidelines.^10^ The control series is constituted of native non‐calcified aortic valves removed from patients who underwent surgery for aortic regurgitation (AR). None of the patients had a diagnosis of infective endocarditis according to the modified Duke criteria.^11^

The primary objective of the study was to compare the occurrence of bacterial detection between CAVD and AR patients. The secondary objectives included the evaluation of bone‐related calcification markers in calcified and non‐calcified cusps, and the comparison of these markers in valve tissues from CAVD and AR patients.

Patient's risk factors evaluated at baseline included gender, age, the diagnosis of bicuspid aortic valve, diabetes, kidney disease, cardiovascular concomitant diseases, cancer, chronic liver disease, chronic kidney disease, smoking habit, anticoagulant or antiplatelet therapy. Markers of lipid metabolism (LDL, HDL, triglycerides), cell blood count and differential and fasting glucose level were evaluated at baseline and correlated with the expression of valve osteogenic markers and total calcium valve content.

The study has been approved by the local Ethic Committee (study approbation number 0025690/23) and has been published on clinicaltrial.gov (NCT05806411).

### 2.2 Valve collection

At the time of surgery, patients' valves were explanted and immediately processed under a sterile hood. Macroscopic evaluation was performed and documented with photographs. Each leaflet was then dissected into four sections within one hour of removal using a scalpel for subsequent microbiological, histological, biochemical and calcium quantification analyses. The three cusps of the valve (or two in case of bicuspid aorta) were grouped according to their anatomy (left coronary LC, right coronary RC, non‐coronary NC); each leaflet was macroscopically described and assigned a consecutive identification code. The description included an evaluation of calcification in the hinge or commissural regions, or both.^12^

Samples for direct and enrichment cultures were immediately processed. Samples for calcium quantification were stored at −80°C, samples for RNA extraction were collected in Qiazol (Qiagen) and stored at −80°C. Samples for bacterial DNA assessment were immediately stored at −80°C before DNA extraction.

Details on sample processing (microbiology, pathology, expression analyses of osteogenic markers, calcium determination, cell culture and VICs staining) are provided in Appendix S1.

### 2.3 Statistical analysis

The sample size was calculated considering a probability of bacterial detection among sampled control of 10%,^13^ a sample of 31 patients and 8 controls could be enrolled to achieve 80% power to detect as significant a proportion of 50% in cases versus the alternative of equal proportions using a chi‐square test with a .05 significance level. The statistical difference in calcium amount and relative expression between pooled data from CAVD and AR leaflets across the three analysed regions was evaluated using a *t*‐test. A *p*‐value of less than .05 was considered statistically significant. As 9 out of 31 patients had a bicuspid aorta, this group of patients was considered in the analysis as a subgroup.

Quantitative markers were compared among groups with multilevel models to take into account the clustered nature of the data with linear regression models having patients and leaflets as random factors. Results are expressed as beta coefficient and 95% confidence interval (95% CI). Dependent variables considered in the analysis were calcium content and expression of early (osterix and COL1A1) and late (ALP, BGLAP) osteoblast markers. Covariates included in the analysis included gender and age, group (cases or controls or bicuspid aorta), aortic leaflet (left coronary or right coronary or non‐coronary), microbiologic findings (leaflets with a PCR result positive for bacterial RNA 16S gene were considered as positive in the analysis), chronic kidney disease, diabetes and dyslipidemia.

## 3 RESULTS

### 3.1 Clinical and demographic characteristics

The study included 31 patients with CAVD and eight controls; among the controls, four had aortic bulb dilatation, two had aortic regurgitation following subaortic ridge resection, one had valvular degeneration, and one had combined mitral–aortic–tricuspid valvular disease. Among the 31 CAVD valves, nine were bicuspid (29%), resulting in 108 valve leaflets, of which 84 were from CAVD and 24 from AR patients. Calcification was mainly localized at the commissural level in 12 leaflets, at the hinge in 21 leaflets and in both sites in 51 leaflets. Localized foci of calcification were randomly observed also in AR patients. No visible sign of infection was observed during surgical procedures either in cases or in controls. Clinical and demographic data are reported in Table 1. Cases and controls were comparable for body mass index (BMI) and clinical characteristics, while dyslipidemia was more common in CAVD than in AR patients (54.8% vs. 12.5%, *p* = .03) and fast glucose level (108 vs. 99.5 mg/dL, *p* = .05) and total WBC (8505 vs. 6540 cells/mmc, *p* = .02) were higher in CAVD than in AR patients.

**TABLE 1:** Epidemiological characteristics of cases and controls.

	Calcific aortic valve disease patients (cases)	Aortic regurgitation (controls)	*p*‐Value
Males, *n* (%)	20 (64.5%)	5 (62.5%)	.9
Age (mean, range)	72 (67–75)	66 (61–79.5)	.7
BMI	27 (24–30)	25 (22–26.5)	.2
Hypertension	23 (74.2%)	6 (75.0%)	1
Diabetes	11 (35.5%)	1 (12.5%)	.2
Chronic kidney disease	2 (6.5%)	1 (12.5%)	.6
Cancer	6 (19.4%)	1 (12.5%)	.7
Chronic liver disease	2 (6.5%)	0	.5
Smoking	10 (32.3%)	1 (12.5%)	.3
Dyslipidemia on treatment	17 (54.8%)	1 (12.5%)	.03
Anticoagulant therapy	6 (19.4%)	2 (25.0%)	.7
Antiplatelet therapy	16 (51.6%)	3 (37.5%)	.5
Metformin	3 (9.7%)	1 (12.5%)	.8
Other hypoglycemic agents	7 (22.5%)	1 (12.5%)	.5
Aortic stenosis	22 (70.9%)	0	.000
Aortic insufficiency	1 (3.2%)	7 (87.5%)	.000
Stenoinsufficiency	8 (25.8%)	1 (12.5%)	.000
Coronary Artery Disease (CAD)	30 (96.8%)	7 (87.5%)	.29
WBC	8505 (6480–9910)	6540 (5325–7440)	.02
Neutrophils	5290 (4180–7130)	3890 (3500–5290)	.12
Lymphocytes	1730 (1450–2510)	1490 (1290–1685)	.06
Blood glucose level	108 (96–135)	99.5 (80–105)	.05
Triglycerides	106 (85–143)	95 (65–134)	.21
Cholesterol	154 (114–215)	148 (128.5–198)	.9

### 3.2 Histopathology

**FIGURE 1:**
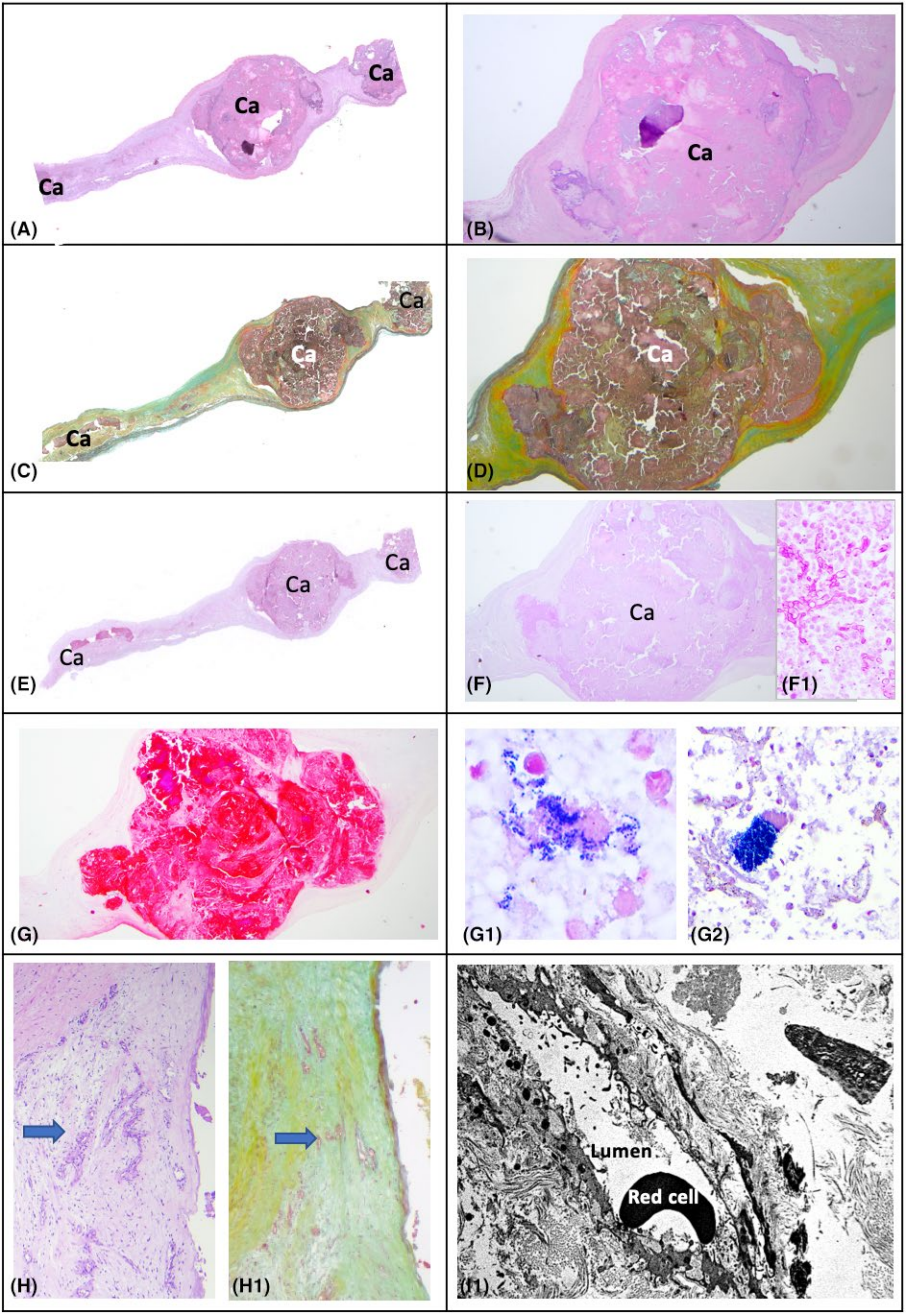
The figure shows the spectrum of major morphological changes occurring in CaVD. Panel (A) H&E stain, low magnification view of a valve sample showing a large nodular calcification (nearly acellular) in the context of the thickness of the leaflet; enlarged in panel (B) which highlights the fibro‐sclerotic context of the calcified nodule. The panel (E) shows the corresponding PAS stain, enlarged in (F), highlighting the absence of PAS‐positive pathogens that, vice versa, stain a positive pneumonia control (F1). The panel (G) shows the same sample staining negative with Gram, vs. Positive Gram controls on bacterial pneumonia (panels G1 & G2). Panel (H) and H1 show H&E‐ and Movat‐stained, non‐calcified area of a calcified leaflet showing micro‐angioneogenesis in the absence of significant inflammation; a feature confirmed also with ultrastructural study (Panel I).

In CAVD samples, light microscopy showed near‐complete loss of the structural integrity of the three layers of the semilunar cusps in both tricuspid and bicuspid valves. In all cases, calcified masses extended into the thickness of the leaflets (Figure 1) or protruded from the leaflet profile. No superficial thrombotic stratifications were observed. GRAM and PAS staining did not reveal the presence of bacteria in any of the leaflets examined. The non‐calcified areas showed structural disruption due to fibrosclerosis or focal, non‐specific myxoid patterns. Cellularity in these areas was sparse and predominantly composed of fibroblasts and smooth muscle cells, with scattered chronic inflammatory cells, mainly macrophages and rare lymphocytes (indicative of very low‐grade inflammation). Focal micro‐angiogenesis was also observed in the non‐calcified areas. Despite the presence of substantial calcium deposits, no areas of cartilage or bone metaplasia were detected.

**FIGURE 2:**
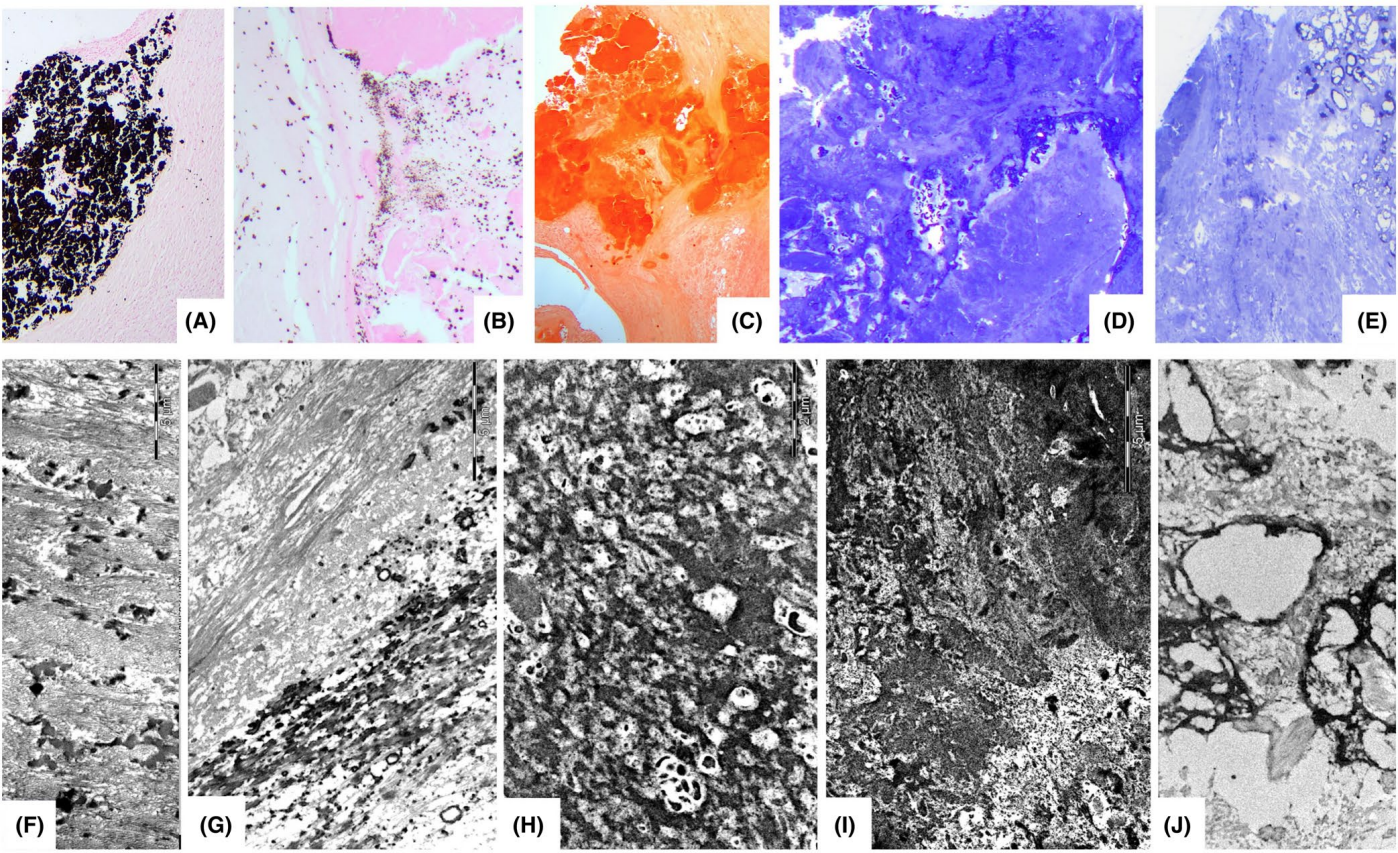
Light microscopy. Spectrum of calcifications observed at light and electron microscopy in the leaflets of CaVD. (A, B): Von Kossa Stain; (C) Alizarin red stain; (D, E): Toluidine blue‐stained semi‐thin section showing the dense calcific masses (dark blue in D) and micro‐calcification (E). Ultrastructural studies. Electron micrographs showing the spectrum of calcifications observed in CaVD leaflets, from microcalcifications scattered within the collagen bundles (F), to calcification of entire bundles of collagen (G), dense reticular calcification ground with microdeposits in spared spaces (H), calcification masses (I) and calcified cellular debris (J).

At transmission electron microscopy (Figure 2) a spectrum of dense, compact, amorphous calcifications and calcified collagen fibrils was variably present in all calcific deposits; calcified bacterial bodies were not observed. Osteoblasts and chondrocytes were also not detected.

**FIGURE 3:**
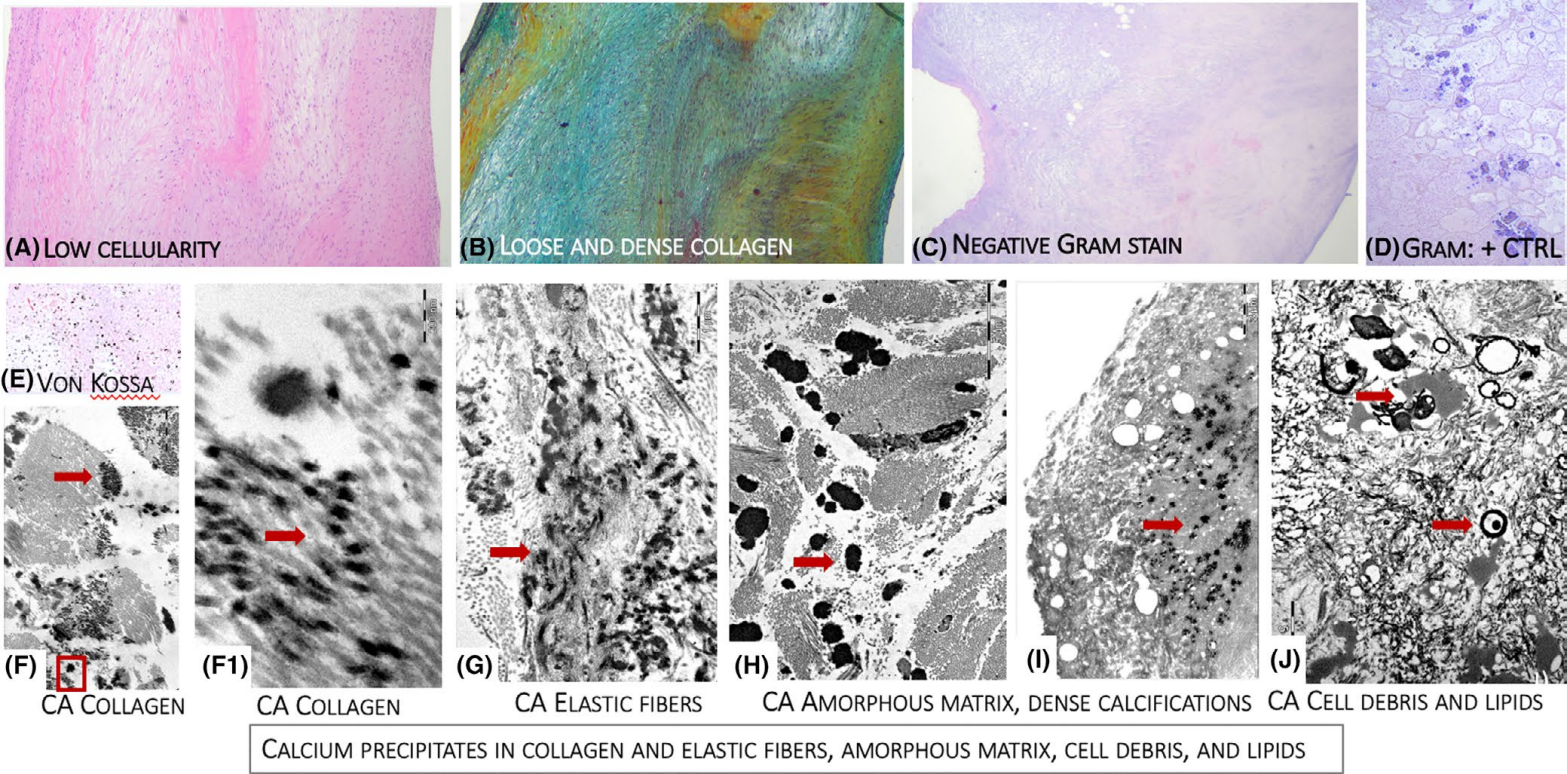
The figure shows the common fibrous remodelling in valves with AR, including loose and dense fibrous tissue within the thickness of the leaflets (A: H&E, B: Movat). Negative Gram‐stained leaflets (C), paired with its corresponding positive control (blue bacterial colonies) from a bacterial pneumonia (D). A common finding in these valves is the presence of microcalcifications detectable with both light (Von Kossa Stain – E) and electron microscopy in panel (F): The red‐squared area Is enlarged in F1), showing early calcium precipitates on single collagen fibrils (red arrow). The panel (G) shows that elastic fibres other than collagen fibrils are common sites of calcium precipitation (all black fibres) (red arrow) (G). Calcium precipitates are also common in the amorphous extracellular matrix, where they appear as smooth‐bordered (H) (red arrow) or irregular‐bordered microdeposits (red arrow) (I). Lipids (red arrow) and residual cell remnants (red arrow) where calcium can precipitate (J). Overall, most microcalcifications result from calcium precipitation in all components of the extracellular matrix.

In AR samples light microscopy (Figure 3) showed remodelling of the valve structure, with dense fibrosis/fibrosclerosis alternating with areas of loose fibrosis, occasional adipocytes and rare lipid droplets. Cellularity was low, consisting mainly of fibroblasts and smooth muscle cells. The sparse presence of inflammatory cells (macrophages and small lymphocytes) was common in all samples. Von Kossa‐positive microcalcifications were variably scattered throughout the cusps. GRAM and PAS staining tested negative. Transmission electron microscopy revealed microcalcifications involving collagen and elastic fibres, amorphous matrix, lipids and cellular debris, with variable density and distribution. No calcified bacteria were identified in the control cusps.

### 3.3 Microbiology

#### 3.3.1 Direct and enrichment culture

Direct culture of all 108 valve leaflets yielded negative results, whereas enrichment culture was positive for five leaflets from 31 CAVD patients (5.95%) and for three leaflets from one single non‐calcific valve out of eight (4.16%). Coagulase‐negative *Staphylococci* were isolated from the enrichment culture of four CAVD leaflets (in two cases *S. warneri* and in other two cases *S. pettenkoferi*), and *Streptococcus oralis* was isolated from the enrichment culture of one CAVD leaflet. Furthermore, *S. oralis* was isolated from the three leaflets of a single AR valve.

#### 3.3.2 16S rRNA sequencing detected low‐virulent bacteria

PCR amplification of the V3‐V5 region of 16S rRNA was positive in 7 of the 31 CAVD valves (22.5%) and in 1 valve (3 leaflets) in the control valves (12.5%) (*p* = .5). In detail, eight leaflets from seven calcific valves (8 out of 84 leaflets, 9.52%) and three leaflets from a single patient in the control group (3 out of 24 leaflets, 12.5%) tested positive. PCR amplification of the V3‐V5 region was negative for all the negative controls, warranting the sterility of the amplification procedure.

Therefore, the V3‐V4 region of 16S rRNA gene of eight CAVD leaflets and three AR leaflets was sequenced. A total of 3,996,077 ASVs were retrieved from sequencing, and after the removal of non‐bacterial ASVs, 3,822,263 ASVs (95.65%) were available for further analysis. The relative abundance of bacterial phyla, classes, orders and families for both cases and controls is shown respectively in Figures S1–S4, respectively. *Firmicutes* was the predominant phylum followed by *Proteobacteria*. The most prevalent families were *Staphylococcaceae*, *Streptococcaceae* and *Enterobacteriaceae*.

**FIGURE 4:**
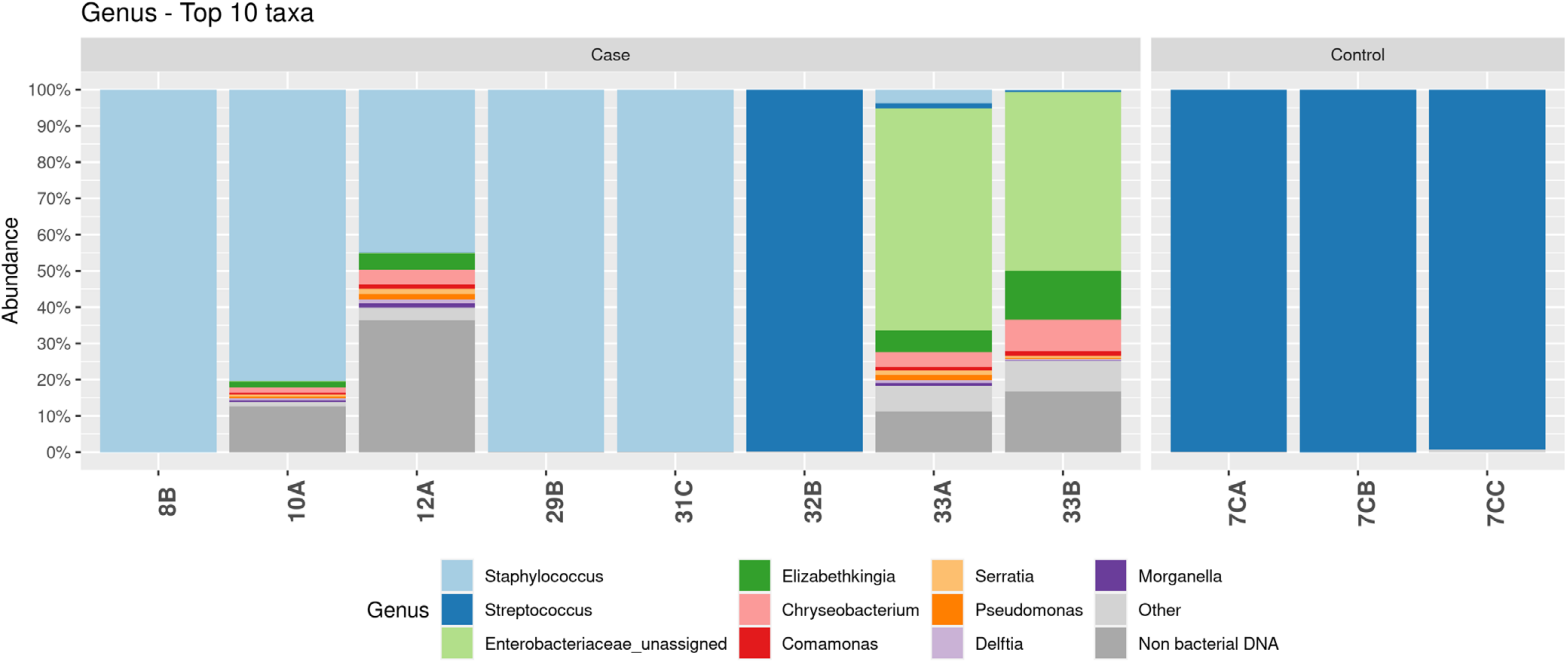
Relative abundances of the taxa at the genus level.

ASVs assigned to *Staphylococcus* spp. were found in 5 CAVD leaflets, in all with relative abundance greater than 80% (Figure 4). For four of these leaflets, *Staphylococcus* spp. was also isolated in the enrichment culture.

ASVs assigned to *Streptococcus* spp. were found in one leaflet of a CAVD valve with a relative abundance of 99.8%. This same leaflet tested positive for *S. oralis* in the enrichment culture.

ASVs assigned to the Enterobacteriaceae family were found in two CAVD leaflets from a single CAVD valve, with relative abundances ranging from 53% to 64%. However, no bacteria belonging to the *Enterobacteriaceae* family were isolated in the enrichment culture.

ASVs assigned to other microorganisms with low pathogenic potential, such as *Elizabethkingia* spp., *Chryseobacterium* spp., *Comamonas* spp., *Serratia* spp., *Pseudomonas* spp., *Delftia* spp. and *Morganella* spp., were found among the ASVs of four leaflets from three valves, with relative abundances between 1% and 15%. In all cases, the leaflets were already considered positive as they had ASV predominance of other bacterial genera (Figure 4).

For the AR valves (controls), three leaflets of a single valve showed *Streptococcus* spp. as the most prevalent genus in ASVs. Concordance between the genus grown in culture and the prevalent genus in the ASVs was observed in this case as well.

### 3.4 Calcium amount is significantly increased in CaVD samples and was associates with dyslipidemia

**FIGURE 5:**
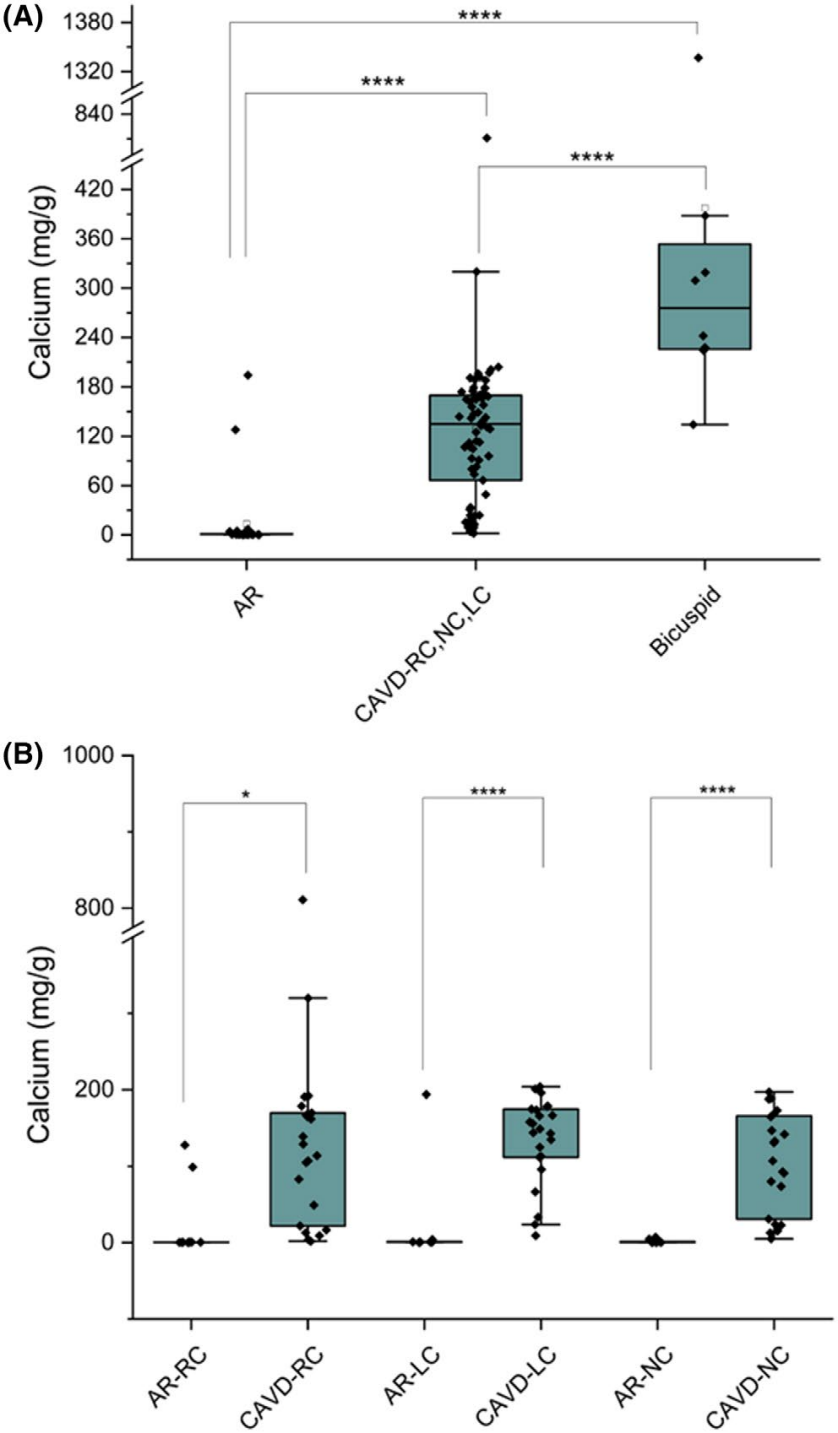
Calcium quantification in control and CAVD human aortic valve leaflets. Calcium levels were measured by ICP‐OES in pooled AR and CAVD valve leaflets (A) and in individual leaflets (right coronary [RC], left coronary [LC] and non‐coronary [NC]) from control and CAVD samples (B). A significant increase in calcium amount is evident in CAVD samples. *p = .052, ****p < .0001.

At univariate analysis, the ICP‐OES analysis revealed a significantly higher calcium amount in CAVD samples compared to AR (coeff. 111, 95% CI 46–176, *p* = .001). Bicuspid aorta leaflets calcium content was increased compared to AR calcium content (coeff. 186, 95% CI 98–273, *p* < .0001). A significant increase was also observed by *t*‐test analysis comparing controls versus CAVD tricuspid aorta valves and CAVD bicuspid aorta valves and specific leaflets in the LC and NC, but not in the RC samples (Figure 5). Moreover, at the univariate analysis, dyslipidemia was associated with increased calcium content (coeff. 74, 95% CI 12.6–137, *p* = .02). At multivariable analysis, factors associated with calcium contents were the CAVD group (coeff. 77, 95% CI 4.2–150, *p* = .04) and bicuspid aorta group (coeff. 167, 95% CI 78–257, *p* < .0001) compared to AR.

In the CAVD subset of patients, valves' calcium content was higher in leaflets with positive microbiologic results, both at univariable (coeff 126, 95% CI 17–236, *p* = .024) and multivariable (coeff 124, 95% CI 4–225, *p* = .043) analysis. The expression of the early osteoblast marker osterix correlates with positive microbiological results.

**FIGURE 6:**
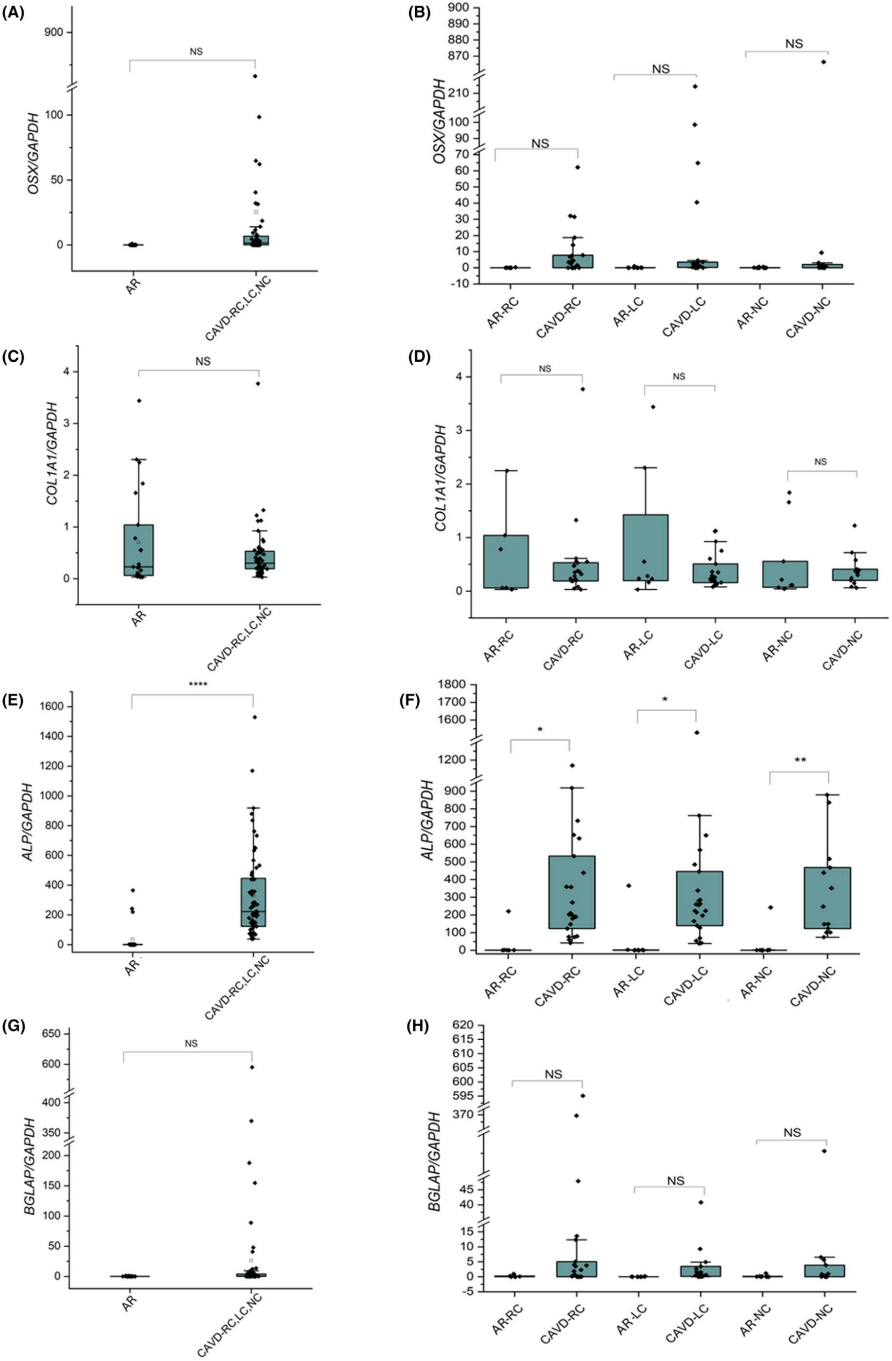
Gene expression analysis of early and late osteoblastic markers. The expression of osterix (*OSX*) (A, B), collagen type I (*COL1A1*) (C, D), alkaline phosphatase (*ALP*) (E, F), osteocalcin (*BGLAP*) (G, H) was analysed in pooled AR and CAVD aortic valve leaflets and in individual leaflets (right coronary [RC], left coronary [LC] and non‐coronary [NC]) from control and CAVD samples. Significant increase in OSX and ALP is evident in CAVD samples.

The expression of early (*osterix* (*OSX*) and *COL1A1*) and late (*ALP, BGLA*P) osteoblast markers were evaluated by qPCR. OSX expression was comparable among leaflets (R: 19.45‐fold, L: 40.6‐fold, and NC: 19.76‐fold) and among CAVD cases and controls (Figure 6). Interestingly, the expression of Osx was significantly higher in leaflets with positive microbiological results both at univariable (coeff. 116, 95% CI 50–182, *p* = .001 95% CI) and multivariable analysis (coeff. 123, 95% CI 58.5–189.2, *p* < .0001). After controlling for leaflet calcification severity, the association between positive microbiologic results and higher OSX expression remained significant at multivariable analysis (coefficient 133; 95% CI 66–201 *p* < .001).

No significant differences in COL1A1 expression were observed between CAVD and AR patients, either overall or when analysing individual valve leaflets (RC, LC and NC) separately (Figure 4B). ALP expression was significantly elevated in CAVD leaflets relative to AR, both overall and within each individual valve leaflet (RC, LC and NC) (Figure 6). However, no association was observed with the presence of positive microbiological findings.

*COL1A1* was increased in younger patients, both a univariable (coeff. −.02, 95% CI −.04–.004, *p* = .02) and multivariable analysis (coeff −.02, 95% CI −.04–.0002 *p* = .05). *BGLAP* expression was comparable among the groups (Figure 4D), it was increased in patients with dyslipidemia at multivariable analysis (coeff. 58, 95% CI 16–101, *p* = .007). ALP expression was increased in CAVD patients compared with AR both at univariable (coeff. 348, 95% CI 169–527, *p* < .0001) and multivariable analysis (coeff. 348, 95% CI 169–527, *p* < .0001) also it was increased in bicuspid aorta patients compared with AR at univariable (coeff. 276.7, 95% CI 91.8–461.6, *p* = .003) and multivariable (coeff. 298.3, 95% CI 105–491, *p* = .002).

### 3.5 Cell culture VICs staining

**FIGURE 7:**
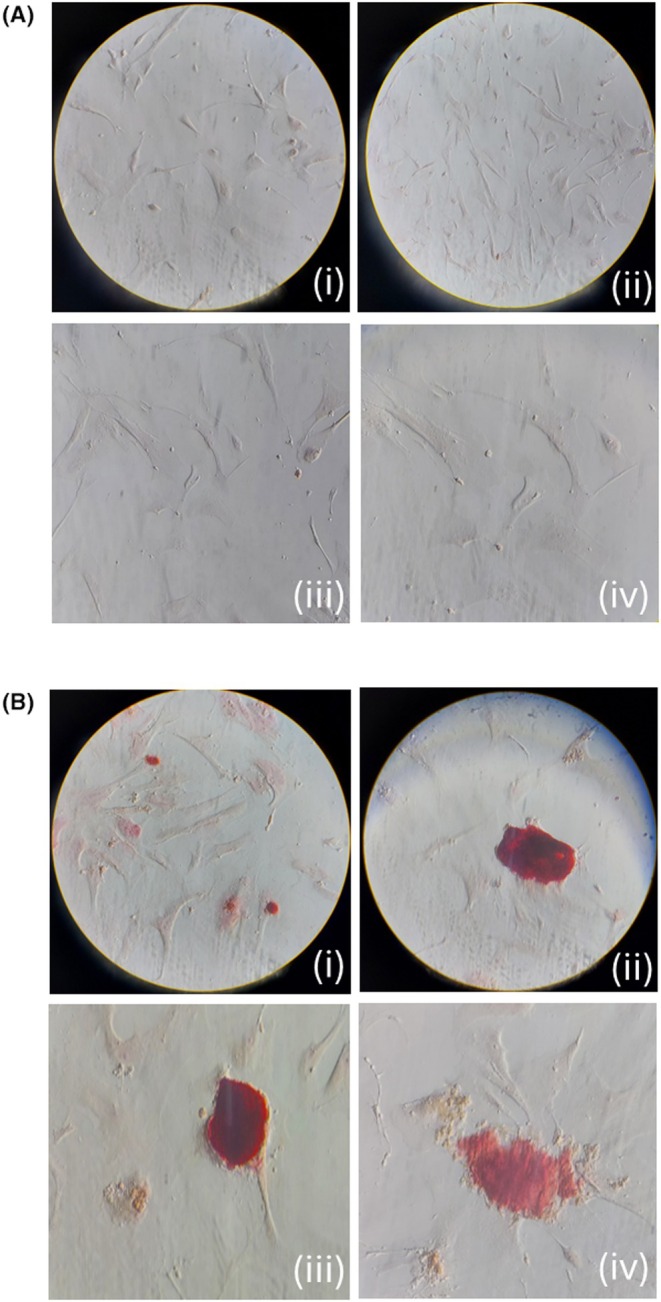
Osteogenic transformation in CAVD VICs cultured without differentiation medium. Valve interstitial cells (VICs) from AR (A) and CAVD (B) patients were cultured in standard (non‐osteogenic) medium. No evidence of mineralization was observed in control VICs, whereas CAVD‐derived VICs exhibited spontaneous osteogenic differentiation, as demonstrated by positive Alizarin Red staining indicating calcium deposition. Ai, Aii 10× magnification; Aiii, Aiv 40× magnification; Bi 10× magnification; Bii, Biii, Biv 40× magnification.

Valve interstitial cells (VICs) isolated from four CAVD and four AR samples were cultured under standard growth conditions, without the addition of osteogenic differentiation media. Remarkably, VICs derived from CAVD patients exhibited spontaneous mineral deposition, as evidenced by positive Alizarin Red staining (Figure 7). In contrast, no mineralization was observed in VICs from control valves under the same conditions. Notably, the formation of calcific nodules was exclusively detected in CAVD patient‐derived cultures. These findings suggest that VICs from CAVD patients possess an intrinsic osteogenic potential, capable of promoting calcium deposition in vitro even in the absence of mineralizing stimuli.

## 4 DISCUSSION

The present study investigated the occurrence of bacterial infiltration in CAVD and in AR valves using histopathology, direct and enrichment cultures and molecular‐based techniques. Histopathology did not provide evidence of bacterial presence or significant differences in tissue inflammation between enrichment‐positive and ‐negative leaflets. Direct culture of all 108 valve leaflets yielded negative results while enrichment culture detected bacterial presence in 5.95% of CAVD leaflets and 4.16% of non‐CAVD leaflets, indicating that some bacteria were viable but at levels too low for direct culturing. In fact, sonication may improve bacterial detection compared to direct culture methods.^14^ Other studies of valves from patients with structural valve diseases^7^, ^8^ and non‐endocarditis cardiac valves^15^ did not find bacterial presence through cultural methods, as in both papers sonication step and enrichment culture were not performed. Thus, one of the purposes of the present project was to couple the challenging culture‐based methods for bacterial characterization of aortic valves with more sensitive culture‐free molecular techniques to improve the efficiency of the diagnosis. Bacteria have been detected only in enrichment culture in roughly 5% of patients, while standard culture yielded negative results. A good correlation was observed between enrichment culture and molecular methods. To attest the sterility of the procedure, negative controls tested negative in the molecular analysis. Bacteria isolated from the enrichment culture included coagulase‐negative *Staphylococci* (*S. warneri* and in other two cases *S. pettenkoferi*) and *Streptococcus oralis*. Both *Staphylococcus* species share the features associated with low virulence species and often harbour genes for biofilm production.^16^, ^17^ *S. oralis* is one of the most abundant commensal bacteria in the human oral cavity, with relatively low pathogenicity and virulence; it retains the ability to produce biofilm and, under favourable circumstances, can become pathogenic.^18^ PCR amplification of the V3‐V5 regions of the 16S identified ASVs assigned to bacteria in 22.5% of CAVD patients and 12.5% of AR patients, while the percentage of positive cusps was overlapping among the two groups (roughly 10%). Indeed, ASVs assigned to *Staphylococcus* spp. and *Streptococcus* spp. were found at high relative abundances in 19.5% of CAVD and 12.5% of AR leaflets. Most of these leaflets also tested positive in enrichment cultures, supporting the presence of viable bacteria. On the other hand, ASVs *of Enterobacteriaceae* were detected in a single CAVD case (two leaflets); however, these bacteria did not grow in culture. This discrepancy may indicate the presence of non‐viable bacterial DNA possibly associated with a transient valve colonization. However, in this study, the frequencies of bacteria ASVs detected in CAVD samples were lower than those previously reported in the literature.^7^, ^8^, ^15^, ^19^ Di Bella and colleagues^15^ found bacterial DNA in 44% of the 34 valves which included 20 aortic and 14 mitral valves. In six out of 15 valve samples, DNA of *Escherichia coli* was found, and DNA of *Enterococcus faecalis* and *Gemella sanguinis* was found in three valve samples each. DNA assigned to other bacteria such as *Acinetobacter baumannii*, *Lysinibacillus fusiformis*, *Mycobacterium* spp., *Pseudomonas aeruginosa*, *Staphylococcus* spp., *Streptoco*ccus spp. and *Stenotrophomonas maltophilia* was found in the remaining samples.

Curini et al.^19^ documented the presence of a CAV‐associated microbiota in 20 patients enrolled in Germany (eleven patients) and Italy (nine patients), 75% of whom showed severe aortic calcification. Oberbach and colleagues^8^ evidenced that 72% of patients with heart valve disease were DNA positive for bacteria commonly associated with IE, such as *C. acne*s (59%), *E. faecalis* (16%), *S. aureus* (15%) and *S. pyogenes* (10%), while bacterial 16S‐rDNA could be detected in 94% of the heart valve disease cohort. The detection of bacterial ASVs in the present study is corroborated by culture methods. When comparing our results with those reported in the literature, Di Bella et al. and Oberbach et al.^8^, ^15^ did not find in the cultures the same bacterial species that they had detected via 16S sequencing, while Curini et al.^19^ did not perform bacterial cultures. However, robust comparisons of our 16S sequencing results with existing literature are challenging due to the lack of harmonization in the methods used. The bacterial genera found through 16S rRNA gene sequencing in our study differed from those reported in previous studies. The identification of distinct bacterial genera in the literature, alongside the detection of *Staphylococcus* and *Streptococcus* species in the valves analysed in our study, raises the question of whether these bacteria play an active role in valve calcification or merely represent incidental colonizers.

Although some genera detected in this study, such as *Comamonas*, *Chryseobacterium* and *Elizabethkingia*, are known to have limited pathogenic potential and have also been reported as reagent contaminants, they were detected in four leaflets (three valves) at low relative abundances. Notably, these taxa were observed in samples characterized by a high relative abundance of a different bacterial taxon. While their biological relevance cannot be entirely excluded, we speculate that bacteria potentially involved in valve calcification would be expected to be present at a predominant biomass. In contrast, low‐abundance taxa may reflect transient colonization or technical contamination and should therefore be interpreted with caution. In a rabbit model, Cohen and colleagues^13^ evidenced that some low virulence bacteria from the mouth may cause subclinical endocarditis that may be associated with valve calcification. Although our study does not establish a definitive causal link between oral microorganisms and aortic valve calcification in humans, it highlights the potential for such an association. In fact, although bacterial detection rates were low, the early osteogenic marker osterix was upregulated in patients whose valves tested positive for microbial presence. These data suggest a potential contributing role for bacteria in driving cellular differentiation towards an osteoblastic phenotype. The increased expression of the early osteoblastic marker osterix is in line with previous reports that found increased levels of OSX adjacent to calcified nodules.^20^ The proximity of Osx‐positive VICs to inflammatory immune cells highlights its central role in inflammation‐driven calcific remodelling. Pattern recognition receptors (PRRs) require identification of microbe‐associated molecular patterns (MAMPs), which include lipopolysaccharide, lipoteichoic acid, lipoprotein and peptidoglycan, in order to trigger host immune responses.^21^ Bone cells are modulated by secretory microbial compounds.^22^ Bacterial infections can directly influence bone homeostasis through stimulating osteoclast differentiation and activation as well as reducing osteoblast activation and differentiation.^23^ Moreover, bacteria and their MAMPs have been reported to influence osteoimmunological responses.^24^ Bacterial components can stimulate osteoclastogenesis and alveolar bone resorption by activating pro‐inflammatory cytokines, including IL‐1β, IL‐6 and TNF‐α.^25^ As a result, immune cells release pro‐inflammatory mediators that activate VICs.^26^ Immunohistochemical investigations of human diseased valves demonstrate high co‐expression of Osx in VICs and inflammatory cells, especially around calcified regions.^20^ The close relationship of Osx positive VICs to inflammatory immune cells emphasizes its essential involvement in inflammation‐driven calcific remodelling.^27^ These findings seem in agreement with the increase of osterix expression in our cohort, in particular in those patients with concomitant bacterial DNA detection.

VICs represent a key factor for progression of CAVD; they can be activated by different stimuli. Previous studies reported that calcified VICs spontaneously develop calcified nodules and exhibit positive staining with Alizarin Red in standard culture, whereas control (healthy) VICs necessitate osteogenic media for this process.^3^, ^28^ VICs derived from calcified valves that exhibited substantial Alizarin Red staining at standard growing conditions imply an activated osteogenic phenotype; however, control VICs are stained only following osteogenic induction. Thus, calcified VICs preserve a persistent osteogenic state, whereas healthy VICs need induction.^29^, ^30^

The main limitations of this study are its single‐center design and the relatively small sample size, which may limit the generalizability and reproducibility of the findings. In particular, all patients included were Caucasian of Italian origin, which may reflect a specific bacterial cluster unique to this population. Moreover, AR patients included as controls showed leaflet degeneration, a condition that may be associated with altered blood flow and potentially enhanced bacterial adhesion, as described for the case with *Streptococcus oralis*.

The strengths of this study lie mainly in the multidisciplinary approach, which ensured a comprehensive analysis of the potential role of bacteria in CAVD pathogenesis.

## 5 CONCLUSIONS

Our study showed that the presence of bacteria in CAVD valves is under the threshold of detection with direct culture and light and electron microscopy study but the combination of enrichment culture with molecular techniques enhances bacterial detection, even at a low rate. The early osteogenic marker osterix was found to be upregulated in patients whose valves tested positive for microbial presence, suggesting a potential role for bacteria in driving cellular differentiation towards an osteoblastic phenotype in CAVD.

## AUTHOR CONTRIBUTIONS

A.F., P.G., C.M.S.R., E.S. were involved in conceptualization. A.A.Q.A., I.M., F.A., S.P. were involved in methodology. A.K., M.C.P.C., T.P., M.F., were in‐volved in data collection and investigation. G.P., A.D.S. and S.R. were involved in data analysis. B.F., were involved in writing original draft. All authors were involved in writing review and editing. G.Z., F.R. and L.F. were involved in visualization. A.A., V.V. and A.S. were in‐volved in supervision. R.B. and E.S. were involved in project administration. E.S. and A.D.S. were involved in funding acquisition.

## CONFLICT OF INTEREST STATEMENT

The authors declare no conflicts of interest.

## Supporting information


Figure S1.

Appendix S1.

## Data Availability

The data supporting the findings of this study are available from the corresponding author upon reasonable request.
