# Supplementary material for: Infectious seeds of valve calcification: Exploring the bacterial hypothesis in the pathogenesis of calcific aortic valve disease
Source: Eur J Clin Invest. 2026 Mar 8;56(3):e70188. doi: 10.1111/eci.70188 (PMC12967713; doi:10.1111/eci.70188)
Supplement: Supplementary file 1 — Figure S1. [file ECI-56-e70188-s001.zip › Figure Captiona.docx]

Supplementary1. Relative abundances of the taxa at the phylum level.

Figure Supplementary2. Relative abundances of the taxa at the class level.

Figure Supplementary3. Relative abundances of the taxa at the order level.

Figure Supplementary4. Relative abundances of the taxa at the family level.
