# Supplementary figures and images for: Infectious seeds of valve calcification: Exploring the bacterial hypothesis in the pathogenesis of calcific aortic valve disease
Source: Eur J Clin Invest. 2026 Mar 8;56(3):e70188. doi: 10.1111/eci.70188 (PMC12967713; doi:10.1111/eci.70188)

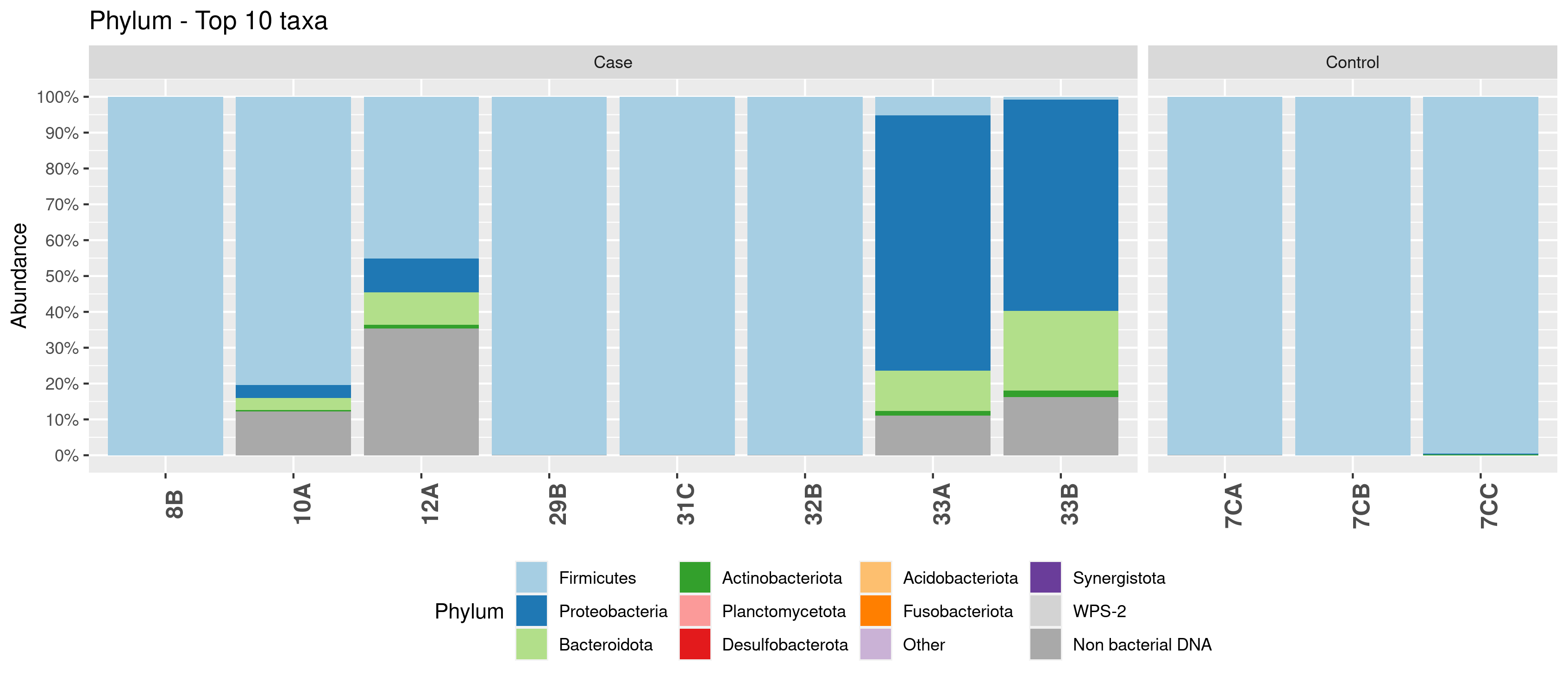

Supplement: Supplementary file 1 — Figure S1. [file ECI-56-e70188-s001.zip › eci70188-sup-0001-FigureS1.png]

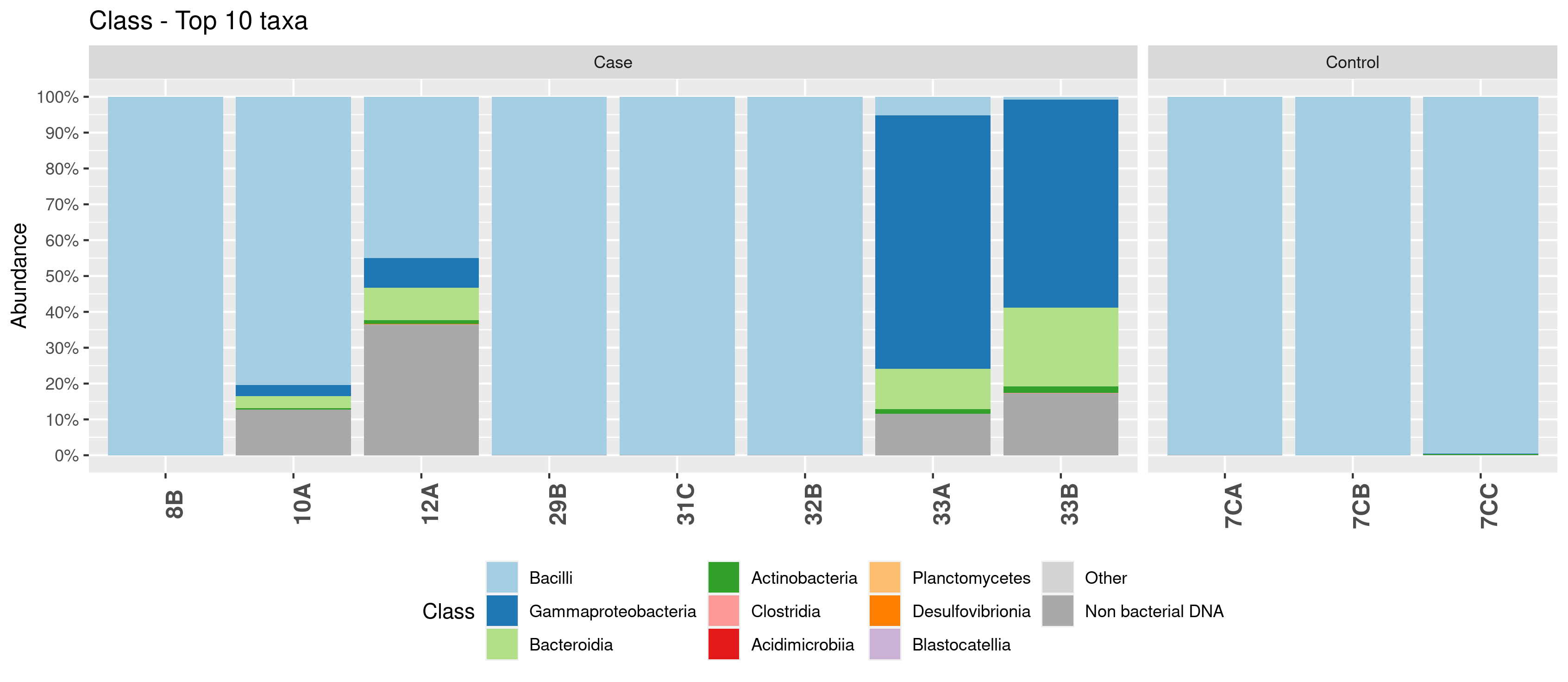

Supplement: Supplementary file 1 — Figure S1. [file ECI-56-e70188-s001.zip › eci70188-sup-0002-FigureS2.png]

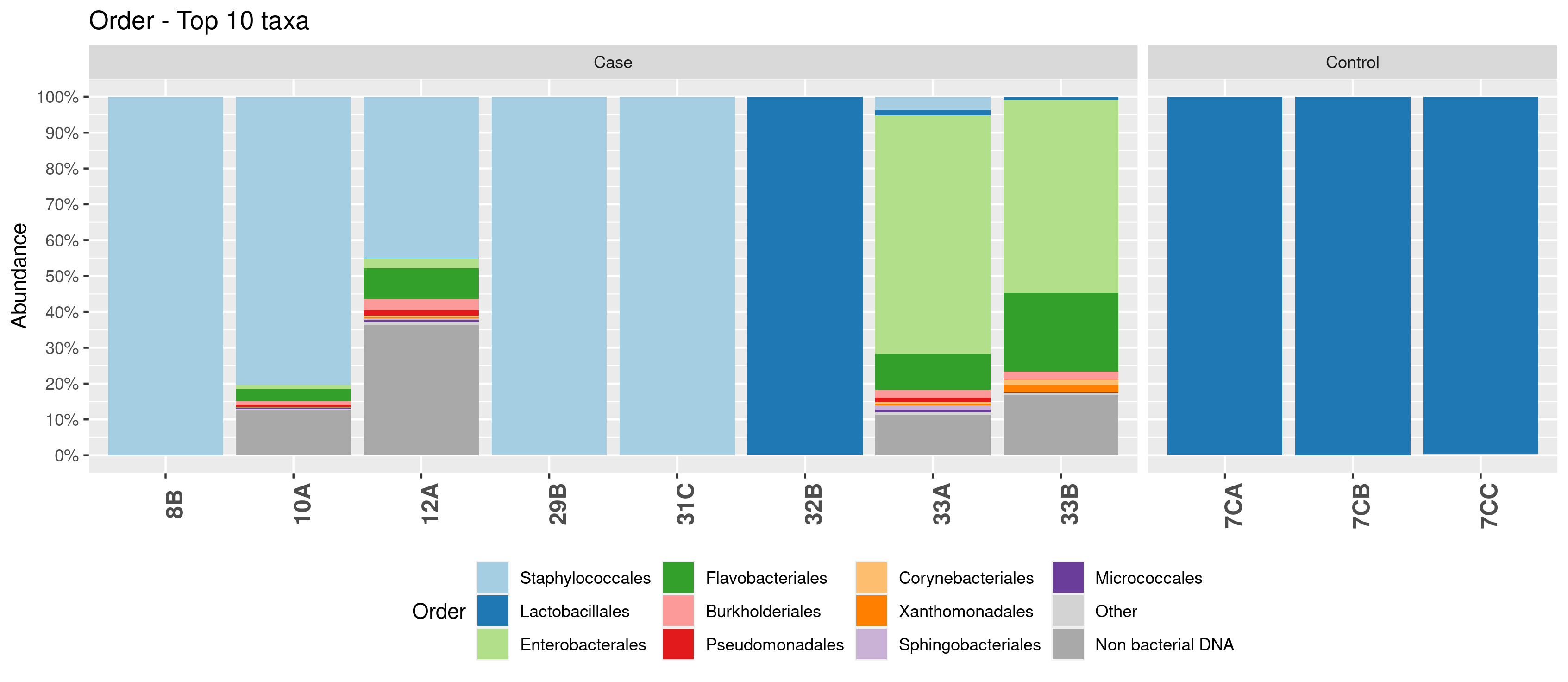

Supplement: Supplementary file 1 — Figure S1. [file ECI-56-e70188-s001.zip › eci70188-sup-0003-FigureS3.png]

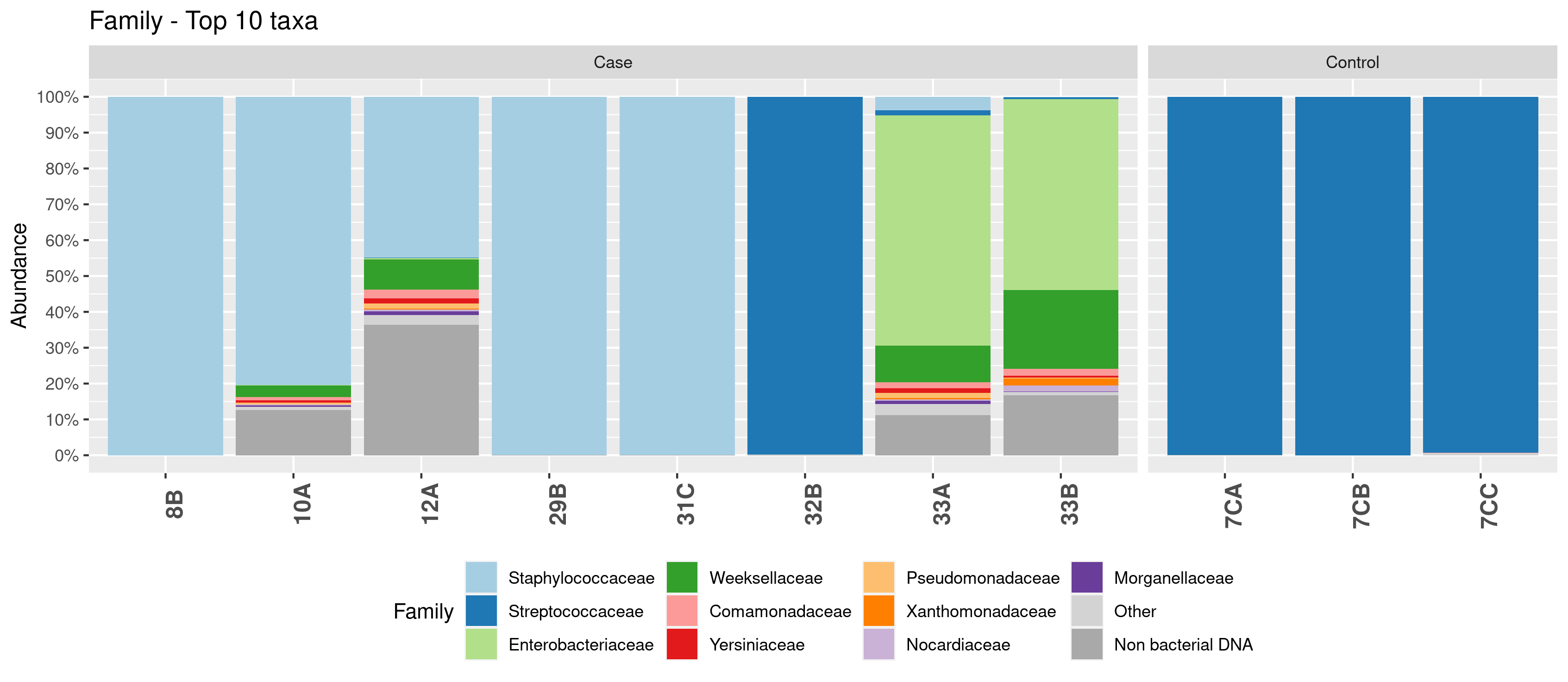

Supplement: Supplementary file 1 — Figure S1. [file ECI-56-e70188-s001.zip › eci70188-sup-0004-FigureS4.png]
