# Supplementary material for: Infectious seeds of valve calcification: Exploring the bacterial hypothesis in the pathogenesis of calcific aortic valve disease
Source: Eur J Clin Invest. 2026 Mar 8;56(3):e70188. doi: 10.1111/eci.70188 (PMC12967713; doi:10.1111/eci.70188)
Supplement: Supplementary file 2 — Appendix S1. [file ECI-56-e70188-s002.docx]

**Pathology study**

For conventional light microscopy histopathology, one sample for each leaflet was immediately fixed in 10% buffered formalin, decalcified, and routinely processed for light microscopy investigation on serially cut tissue sections. Stains for paraffin sections included H&E, MOVAT pentachrome, Von Kossa, and Alizarin Red. The pathology procedures were planned and performed using the same protocols institutionally adopted for clinical evaluation of tissue sample bacterial infections, which include Gram and Periodic Acid-Schiff (PAS) stains as baseline and possible additional stains in case of positive Gram stains. Pathology studies addressed the following issues: characterization of the pathological changes of the 3 layers of the aortic valve (fibrous, spongiosa, and ventricular); exclusion of thrombosis and endocarditis; search for Gram-positive and Gram-negative bacteria. For ultrastructural studies, random fragments of the calcified and non-calcified areas from each sample were collected for electron microscopy. Non-decalcified samples (about 2 mm) were fixed in Karnovsky's solution in cacodylate buffer 0.2 M (pH 7.3) for 4 hours at 4°C; then post-fixed with 1% osmium tetroxide in cacodylate buffer 0.2 M (pH 7.3) for 1 hour at RT, dehydrated in ethanol and propylene oxide, and embedded in epon-araldite resin. Semi-thin sections were stained with toluidine blue, and ultrathin sections were stained with uranyl acetate and Reynolds's lead citrate and observed with a JEOL JEM 1011 electron microscope.

**Microbiology of valves**

All samples for cultures were immediately sonicated (35 kHz) with dithiothreitol for 15 minutes and vortexed for 30 seconds and then plated into Columbia agar +5% sheep blood, Chocolate agar + PolyViteX™, Mannitol Salt agar, MacConkey agar, and Schaedler agar + 5% sheep blood (bioMérieux SA, Marcy-l’Etoile, France). An aliquot of the sonicate was enriched in Brain Heart Infusion broth (COPAN, Brescia, Italy). For bacterial DNA assessment, human DNA depletion was achieved by incubation of the aortic leaflet samples in PBS with 0.0125% saponin (Thermo Scientific, Monza, Italy) for 30 minutes at 37°C in a shaking incubator (400 rpm) in presence of sterile iron beads and successively by incubation with Salt Active Nuclease (Merck, Milan, Italy) for 30 minutes at 37°C, following Bruggeling et al. recommendation (Bruggeling CE, Garza DR, Achouiti S, Mes W, Dutilh BE, Boleij A. Optimized bacterial DNA isolation method for microbiome analysis of human tissues. Microbiologyopen. 2021 Jun;10(3):e1191. doi: 10.1002/mbo3.1191. PMID: 34180607; PMCID: PMC8208965). Genomic DNA was then extracted using the DNeasy PowerSoil Pro Kit (Qiagen, Hilden, Germany) after a treatment with lysozyme (Merck, Milan, Italy) 20 mg/ml. To monitor potential contamination during nucleic acid extraction, two negative control samples, consisting of sterile DNase/RNase-free water, were extracted using the same method, conditions, and kit as the experimental samples. PCR amplification of the V3-V5 variable regions of the 16S rRNA gene was used to screen for the presence of bacterial DNA in each sample. The PCR amplification protocol was performed according to the method described by Regueira-Iglesias et al. (Regueira-Iglesias A, Vázquez-González L, Balsa-Castro C, et al. In-Silico Detection of Oral Prokaryotic Species With Highly Similar 16S rRNA Sequence Segments Using Different Primer Pairs. Front Cell Infect Microbiol. 2022 Feb 9;11:770668. doi: 10.3389/fcimb.2021.770668. PMID: 35223533; PMCID: PMC8863748). The V3-V4 variable regions of the 16S rRNA gene were then sequenced for all samples that tested positive for PCR amplification. Sequencing was performed using a paired-end approach (2×300 cycles) on the Illumina MiSeq platform, following the Illumina 16S Metagenomic Sequencing Library Preparation protocol. The raw sequence data were processed using QIIME2 (version 2022.8) (Bolyen E, Rideout JR, Dillon MR, et al. Reproducible, interactive, scalable and extensible microbiome data science using QIIME 2. Nat Biotechnol. 2019 Aug;37(8):852-857. doi: 10.1038/s41587-019-0209-9. Erratum in: Nat Biotechnol. 2019 Sep;37(9):1091. doi: 10.1038/s41587-019-0252-6. PMID: 31341288; PMCID: PMC7015180.) and the DADA2 algorithm (Callahan BJ, McMurdie PJ, Rosen MJ, Han AW, Johnson AJA, Holmes SP. DADA2: High-resolution sample inference from Illumina amplicon data. Nat Methods. 2016 May 23;13(7):581–3.)  was used for read trimming, low-quality read removal, denoising, run-specific error rate estimation, and inference of amplicon sequence variants (ASVs). Quality control within the algorithm included both detection and removal of chimaeras, eliminating sequence errors, and excluding singletons. ASVs were assigned to taxonomy by training a Naive Bayes classifier with the SILVA 138 database (Quast C, Pruesse E, Yilmaz P, et al. The SILVA ribosomal RNA gene database project: improved data processing and web-based tools. Nucleic Acids Res. 2013 Jan;41(Database issue):D590-6). ASVs assigned to Mitochondria and Eukarya were manually excluded from the analysis and the sample composition was evaluated using the phyloseq package ( McMurdie PJ, Holmes S. Phyloseq: an R package for reproducible interactive analysis and graphics of microbiome census data. PLoS One. 2013 Apr 22;8(4):e61217. doi: 10.1371/journal.pone.0061217. PMID: 23630581; PMCID: PMC3632530.) in R.

**Calcium determination**

Valve samples were thawed, and after weighing, they were placed into glass tubes. Then, 200 µl of Trace-SELECT® Ultra ultrapure HNO_3_ (65 % w/w, Merk, Milan, Italy) and 500 µl of H_2_O_2_ (30 % w/w) were added. Tubes were incubated in a warm ultrasonic bath for 15 min, without drying. At the end, purified water produced by a Milli-Q Direct system (Merck Millipore, Burlington, Massachusetts – USA) was added up to 4 mL. Depending on the calcification status, the time of processing in the ultrasonic bath was increased up to 60 min until tissue debris dissolved and a clear solution was obtained. Three blank samples (200 μl of HNO_3_ and 500 μl of H_2_O_2_) were prepared in parallel and treated in the same way as the test samples. Calcium was determined in each sample by ICP-OES iCAP 7400 Duo (Thermo Fisher Scientific Inc., Waltham – USA), equipped with a concentric nebuliser, a cyclonic spray chamber and a ceramic duo-torch, according to the operating conditions suggested by the manufacturer. An ASX-560 autosampler (Teledyne CETAC, Omaha, Nebraska – USA) was used to transfer samples to the introduction system of the ICP-OES. Thermo Scientific Qtegra™ software ver.2.8.2944.202 was used for data acquisition. Calcium content was normalized for the sample weight and expressed as mg of calcium per g of tissue.

**Expression analyses of the osteogenic markers**

Of the total of 108 samples, 84 samples were considered for osteogenic markers analyses while 24 samples were excluded due to poor RNA quality and degradation. For total valve RNA extraction, tissue samples frozen in 1 ml of Trizol were thawed for 10 min on ice with occasional inversion. Then, each sample was fragmented and homogenized gently in a cool condition on ice and RNA was extracted following the manufacturer recommendations. Total RNA concentration and quality were determined using the 2100 Bioanalyzer chip (Agilent, RIN:4-10). To eliminate genomic DNA contamination, DNase treatment was performed by Turbo DNA Free Kit (AppliedBio-systems) following manufacturer instructions. RNA (1 μg) was reverse transcribed using cDNA Synthesis Kit (High-Capacity cDNA Transcription kit, Applied Biosystems) in a final volume of 30 μL following the protocol described in the instruction manual. RT-qPCR for *RUNX2, OSX, ALP, COL1A1, BGLAP* and *GAPDH* was performed in 25 μL reaction mixtures using SYBR Green Master mix (Applied Biosystems). The QuantStudio 3 thermocycler and the QuantStudio Design and analysis software (Applied Biosystems) were used. The relative expression level of each gene was calculated using the ΔΔCt method (Silvia, et al. 2020). Primer sequences are available upon request.

**Cell culture and VICs staining**

Immediately upon receipt of the samples, four pieces of aortic valve cusps from CAVD cases (n=4) and AR samples (n=4) were cultured, for valve interstitial cells (VICs) isolation using M199 (Sigma Aldrich, USA, D5652) and 10% FBS. For further VICs sub-culturing, medium M199 (Euroclone, Celbio, Milan, Italy) and 10% FBS was used. Primary VICs at P0-P1 were fixed and stained after 10 days of culture with 2% Alizarin red (Sigma Aldrich, China, A5533) to evaluate calcium deposition. Briefly, cells were fixed in 10% formalin (Sigma Aldrich, China, HT5014) for 15 minutes and washed twice with distilled water. After adding Alizarin red solution, cells were stained for 15 minutes at room temperature. Alizarin red stain was visualized by DM IL LED microscope (Leica Microsystems Srl, Buccinasco, Italy).
